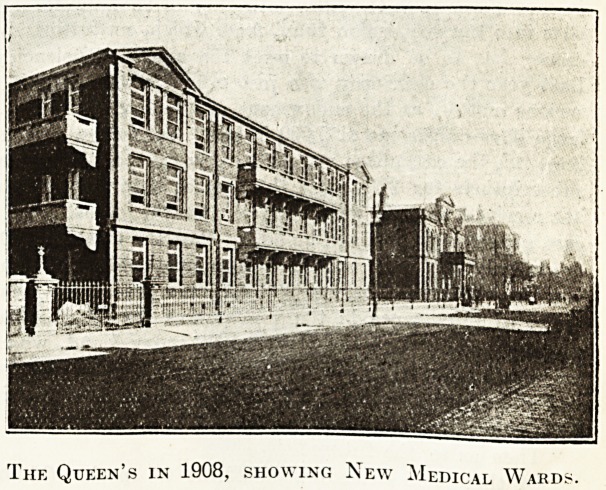# The Evolution of a City Hospital

**Published:** 1912-09-28

**Authors:** 


### THE EVOLUTION OF A CITY HOSPITAL.

### Mr. Arthur Hulme on the Queen's Hospital, Birmingham.

The presence of so large a number of hospital officials in Birmingham last week at the Conference of the British Hospitals Association has inevitably concentrated the interest of the hospital world on the institutions of the Midland capital, and led our Commissioner to seek from Mr. Arthur Hulme, the secretary and general superintendent of the Queen's Hospital, some details of the development of this typical Birmingham institution.

"Though the history of the Queen's," Mr. Hulme began as we started our inspection at the old section of the buildings, "has never been formally written or collected in a single volume, there is more than one summary available of the chief events in its history since it was founded in 1840. Indeed, the report for 1909, the first complete year since the opening of the new medical wards in October 1908, contains a special appendix which summarises its development, in which three crucial dates stand out prominently—that in which the hospital was founded, namely, 1840; the year in which the out-patient department was built, 1873; and 1908, when the new medical wards were opened. The last date, as a glance at the map will show you, marks our matured growth, for as far as a self-contained site is concerned we have now absorbed

The Queen's Hospital in 1841.

all the contiguous ground available, and so for the present at any rate reached the limit of our extension within what I may call the natural boundaries of Bath Row, the Canal, and Falconer Road. Following the general law of human progress we have moved from east to west over the site. The development of the hospital has thus been logical and is very easy to follow. The old block in which we are now, and which at present is devoted to surgical cases, was the original hospital; next to this, a little further west, is the out-patient department, which was added in 1873, and which marked the development of the hospital when I became its secretary ten years later. In 1889 our first nurses' home was built. Then followed a pause of nearly twenty years before the great undertaking of the new medical wards, the chapel, and the dispensary was thrown open. For completeness' sake it should be added that the nurses' home was extended in this year also, when the great boiler-house, comprising an installation of the system of vacuum heating by atmospheric steam, was added."

Mr. Hulme then led the way upstairs from his office, and we inspected the male ward of the old building on the first floor, in a bay of which he pointed out the four beds for ophthalmic patients.

"How many beds are there in this part of the building?"

"Ninety-four, all of which are for surgical cases. Here

The Queen's in 1873, showing Extension.

also are theatres. They have been modernised, as you see; but it has been proved here, as in many other institutions, that good surgical results do not depend upon, or are in any sense guaranteed by, the tiled palaces which were the vogue of ten years ago."

"We are rather proud of our out-patient department," Mr. Hulme continued as we descended the stairs. "Although it was built in 1873 and only slight additions were made to it in 1908, it has served as a model to many institutions. You will note that the unit consists of a central hall, a registration office, examination and consultation rooms, casualty room, and theatre for minor operations. Above these rooms on one side are the assistant matron's apartments and the rooms of several of the domestic staff, and on the opposite side of the waiting-

The Queen's in 1908, showing New Medical Wards.

hall are the quarters of the resident medical and surgical staff."

"Have you an almoner?"

"Not at the present time, though the matter is still under consideration. It is felt that such an officer, if appointed, must not merely assist the patients by supplementing their hospital treatment through other agencies, but must also act as an inquiry officer and undertake that examination of the claims of doubtful persons to free treatment, much of which is now done in the secretary's office. In fact, the provision of sanatorium notes, clothing, and so forth is already secured through the Queen's Hospital Aid Society for in-patients only. At present it would be premature to say what decision may be arrived at on the question."

On leaving the out-patient department we passed on to the dispensary, in charge of Mr. Henry Campbell, M.P.S., which is that part of the 1908 extension to be first approached from the main building.

"The wards here," Mr. Hulme said, "are large in size and have an aggregate of sixty-six beds, the large male and female wards containing twenty-one and twenty-four beds respectively. The ward on the second floor has been divided to accommodate nine men and fifteen children. Apart from the vacuum heating I have already mentioned, you will note the central Shorland stoves, which contrast with the old open grates that you saw just now in the wards of the old main building. The floors are teak, and those of the theatres terrazzo. Then you must also see the roof ward, of which by the way The Hospital published a photograph on December 16, 1911. It was in one sense an after-thought, but was hailed at the time as the first attempt in Europe to treat acute cases in the open air. The demand on its six beds is very great. To-day, as it happens, it is being cleaned, and hopes are expressed that it will be ready by nightfall. It is used for both medical and surgical cases, the men and the women taking it in turn for allotted periods of four months. Under its covered part is the kitchen and annexes, which make this a complete unit. No difficulty has been experienced in serving the meals hot, and the jacketed hot-water tins serve their purpose quite satisfactorily."

"What was the cost of this extension?"

"£45,000; and the hospital was able to make a strong point at the time by reminding the public that the occasion was the only one since the opening of the hospital on which any considerable sum of money had been asked for by the committee. Talking of financial matters, I may say that half the Roderick bequest has been received, and in accordance with the financial policy of the hospital will pass into the suspension fund, from which, unfortunately, money has to be drawn to meet the annual deficiencies. Last year the deficiency was just over £5,000. This is a serious matter, as the endowment of the hospital is not a large one—only some £40,000 in fact. As to the support received, the committee was glad to record that the annual subscriptions for 1911 were the largest that the hospital has received since it was founded, though, in justice to a past generation, it should be added that the total—£3,402—was only £27 more than had been received in 1875, the year in which the hospital was declared free. The next factor of importance in the income was the £2,100 that was received through the Hospital Saturday Fund, which represents a widely diffused sympathy with the hospital on the part of thousands of working men and women in Birmingham."

"Then as to the future?"

"Of that it is exceedingly difficult to speak. Of the two factors of the situation one is national, the other local. The national factor is the Insurance Act, on which my committee decline to commit themselves beyond affirming that it will not reduce the number of in-patients, because it makes no provision for the treatment of serious illness, and as to out-patients their numbers will hardly be reduced since the majority of them are married women or children or other uninsured persons. However, perhaps the Conference of the British Hospitals Association will have some further light to throw upon the matter. The cost of the Insurance Act to the Queen's Hospital at least is certain; it will be about £130 per annum."

"What is the local factor you referred to?"

"The movement to bring about some closer and more permanent co-operation between the Birmingham hospitals. As to how this will be brought about I am quite unable to say. But the Insurance Act and the question of medical certificates to school children have certainly served to bring the Birmingham hospitals closer together. Conferences have been held between representatives of the various institutions, and my committee sincerely hopes that before long some scheme of co-operation will be adopted."

"On the lines of King Edward's Hospital Fund for London?"

"I am not in a position to say. The provinces and the Metropolis have naturally different ways of doing things, and the conditions are so different that in any case it would be unlikely that what suited the one would be suitable to the other. The general feeling, however, is in favour of a closer connection, and, such being the case, it is probable that agreement may be come to on lines acceptable to the different institutions."

## Figures and Tables

**Figure f1:**
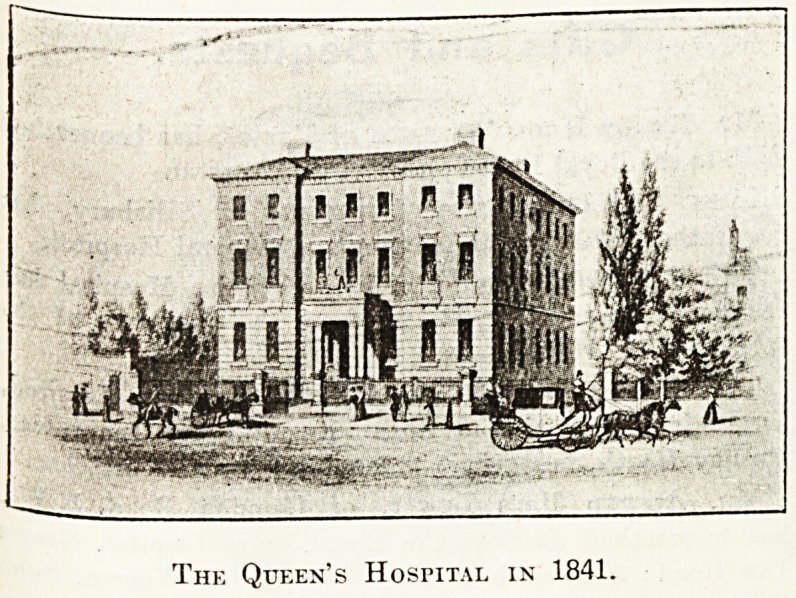


**Figure f2:**
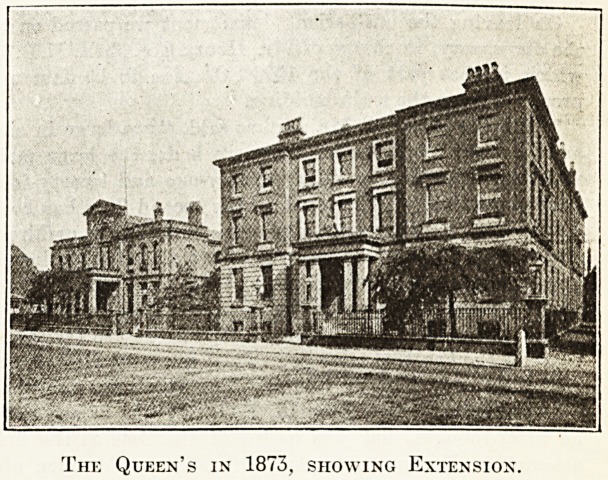


**Figure f3:**